# Finite-time scaling in local bifurcations

**DOI:** 10.1038/s41598-018-30136-y

**Published:** 2018-08-06

**Authors:** Álvaro Corral, Josep Sardanyés, Lluís Alsedà

**Affiliations:** 1 Centre de Recerca Matemàtica, Campus de Bellaterra, Edifici C, 08193 Bellaterra, Barcelona Spain; 2 Barcelona Graduate School of Mathematics, Campus de Bellaterra, Edifici C, 08193 Bellaterra, Barcelona Spain; 3 Departament de Matemàtiques, Universitat Autònoma de Barcelona, Bellaterra, Barcelona Spain; 4 Complexity Science Hub Vienna, Josefstädter Straβe 39, 1080 Vienna, Austria

## Abstract

Finite-size scaling is a key tool in statistical physics, used to infer critical behavior in finite systems. Here we have made use of the analogous concept of finite-time scaling to describe the bifurcation diagram at finite times in discrete (deterministic) dynamical systems. We analytically derive finite-time scaling laws for two ubiquitous transitions given by the transcritical and the saddle-node bifurcation, obtaining exact expressions for the critical exponents and scaling functions. One of the scaling laws, corresponding to the distance of the dynamical variable to the attractor, turns out to be universal, in the sense that it holds for both bifurcations, yielding the same exponents and scaling function. Remarkably, the resulting scaling behavior in the transcritical bifurcation is precisely the same as the one in the (stochastic) Galton-Watson process. Our work establishes a new connection between thermodynamic phase transitions and bifurcations in low-dimensional dynamical systems, and opens new avenues to identify the nature of dynamical shifts in systems for which only short time series are available.

## Introduction

Bifurcations separate qualitatively different dynamics in dynamical systems as one or more parameters are changed. Bifurcations have been mathematically characterized in elastic-plastic materials^1^, electronic circuits^2^, or in open quantum systems^3^. Also, bifurcations have been theoretically described in population dynamics^4–6^, in socioecological systems^7,8^, as well as in fixation of alleles in population genetics and computer virus propagation, to name a few examples^9,10^. More importantly, bifurcations have been identified experimentally in physical^11–14^, chemical^15,16^, and biological systems^17,18^. The simplest cases of local bifurcations, such as the transcritical and the saddle-node bifurcations, only involve changes in the stability and existence of fixed points.

Although, strictly speaking, attractors (such as stable fixed points) are only reached in the infinite-time limit, some studies near local bifurcations have focused on the dependence of the characteristic time needed to approach the attractor as a function of the distance of the bifurcation parameter to the bifurcation point. For example, for the transcritical bifurcation it is known that the transient time, *τ*, diverges as a power law^19^, as *τ* ~ |*μ* − *μ*_*c*_|^−1^, with *μ* and *μ*_*c*_ being the bifurcation parameter and the bifurcation point, respectively, while for the saddle-node bifurcation^20^ this time goes as *τ* ~ |*μ* − *μ*_*c*_|^−1/2^ (see^12^ for an experimental evidence of this power law in an electronic circuit).

Thermodynamic phase transitions^21,22^, where an order parameter suddenly changes its behavior as a response to small changes in one or several control parameters, can be considered as bifurcations^23^. Three important peculiarities of thermodynamic phase transitions within this picture are that the order parameter has to be equal to zero in one of the phases or regimes, that the bifurcation does not arise (in principle) from a simple low-dimensional dynamical system but from the cooperative effects of many-body interactions, and that at thermodynamic equilibrium there is no (macroscopic) dynamics at all. Non-equilibrium phase transitions^24,25^ are also bifurcations and share these characteristics, except the last one. Particular interest has been paid to second-order phase transitions, where the sudden change of the order parameter is nevertheless continuous and associated to the existence of a critical point.

A key ingredient of second-order phase transitions is finite-size scaling^26,27^, which describes how the sharpness of the transition emerges in the thermodynamic (infinite-system) limit. For instance, if *m* is magnetization (order parameter), *T* temperature (control parameter), and $\ell$ system’s size, then for zero applied field and close to the critical point, the equation of state can be approximated as a finite-size scaling law,

1 $$m\simeq \frac{1}{{\ell }^{\beta /\nu }}g[{\ell }^{1/\nu }(T-{T}_{c})],$$

with *T*_*c*_ the critical temperature, *β* and *ν* two critical exponents, and *g*[*y*] a scaling function fulfilling *g*[*y*] ∝ (−*y*)^*β*^ for *y* → −∞ and *g*[*y*] → 0 for *y* → ∞.

It has been recently shown that the Galton-Watson branching process (a fundamental stochastic model for the growth and extinction of populations, nuclear reactions, and avalanche phenomena) can be understood as displaying a second-order phase transition^28^ with finite-size scaling^29,30^. In a similar spirit, in this article we show how bifurcations in one-dimensional discrete dynamical systems display “finite-time scaling”, analogous to finite-size scaling with time playing the role of system size. We analyze the transcritical and the saddle-node bifurcations for iterated maps and find analytically well-defined scaling functions that generalize the bifurcation diagrams for finite times. The sharpness of each bifurcation is naturally recovered in the infinite-time limit. The finite-size behavior of the Galton-Watson process becomes just one instance of our general finding for the transcritical bifurcation. And as a by-product, we derive the power-law divergence of the characteristic time *τ* when *μ* is kept constant, off criticality^19,20^.

## Universality of Convergence to Attractive Fixed Points

In this paper, we consider a one-dimensional discrete dynamical system, or iterated map, *x*_*n*+1_ = *f*(*x*_*n*_), where *x* is a real variable, *f*(*x*) is a univariate function (which will depend on some non-explicit parameters) and *n* is discrete time. It is assumed that the map has an attractive (i.e., stable) fixed point at *x* = *q*, for which *f*(*q*) = *q*, with |*f* ′(*q*)| < 1, where the prime denotes the derivative^20^. Moreover, the initial condition, *x*_0_, is assumed to belong to the basin of attraction of the fixed point. Additional conditions on *x*_0_ will be discussed below.

We are interested in the behavior of *x*_*n*_ = *f* ^*n*^(*x*_0_) for large but finite *n*, where *f* ^*n*^(*x*_0_) denotes the iterated application of the map *n* times. Naturally, for sufficiently large *n*, *f* ^*n*^(*x*_0_) will be close to the attractive fixed point *q* and we will be able to expand *f*(*f* ^*n*^(*x*_0_)) around *q*, resulting in

2 $$\begin{array}{rcl}{f}^{n+1}({x}_{0}) & = & f({f}^{n}({x}_{0}))=q+M({f}^{n}({x}_{0})-q)\\  &  & +\,C{({f}^{n}({x}_{0})-q)}^{2}+{\mathscr{O}}{(q-{f}^{n}({x}_{0}))}^{3},\end{array}$$

with

$$M=f^{\prime} (q)\,{\rm{and}}\,C=\frac{f^{\prime\prime} (q)}{2}.$$

By rearranging and introducing the variable *c*_*n*+1_, the inverse of the “distance” to the fixed point at iteration *n* + 1, we arrive at

$${c}_{n+1}=\frac{1}{q-{f}^{n+1}({x}_{0})}=\frac{{c}_{n}}{M}+\frac{C}{{M}^{2}}+{\mathscr{O}}(q-{f}^{n}({x}_{0})),$$

(we may talk about a distance because we calculate the difference in such a way that it is always positive). Iterating this transformation $\ell$ times leads to

$${c}_{n+\ell }=\frac{{c}_{n}}{{M}^{\ell }}+\frac{C(1-{M}^{\ell })}{{M}^{\ell +1}(1-M)},$$

where only the lowest-order terms have been considered^29^. When the variable *z*, defined as $z={\ell }^{\alpha }(M-1)$, is kept finite (with $\ell \to \infty$ and *M* → 1) a non-trivial limit of the previous expression exists if *α* = 1. It is found that the right-hand side of the expression is dominated by the second term, which grows linearly with $\ell$. Therefore, for large $\ell$, we arrive at ${c}_{n+\ell }\simeq C\ell ({e}^{z}-1){e}^{-z}/z$, and taking the inverse, we obtain

$$q-{f}^{\ell +n}({x}_{0})=\frac{1}{{c}_{\ell +n}}\simeq \frac{1}{C\ell }G(z),$$

with scaling function

3 $$G(z)=\frac{z{e}^{z}}{{e}^{z}-1}.$$

Observe that the sequence ${\{{f}^{\ell }({x}_{0})\}}_{\ell =1}^{\infty }$ is convergent and thus, for $\ell$ large enough with respect to *n*, ${f}^{\ell +n}({x}_{0})\simeq {f}^{\ell }({x}_{0})$. Consequently,

4 $$q-{f}^{\ell }({x}_{0})\simeq \frac{1}{C\ell }G(z).$$

This is exactly the same result as the one derived in ref.^29^ for the Galton-Watson model, leading to the realization that this model is governed by a transcritical bifurcation (but restricted to a fixed initial condition *x*_0_ = 0).

**Figure 1:**
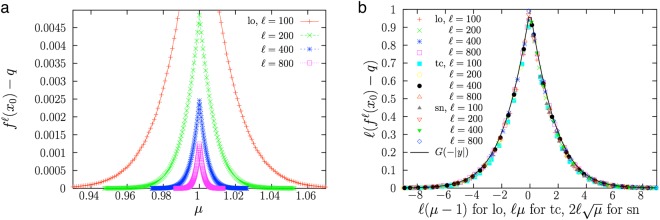
(**a**) Distance between the $\ell$–th iteration of the logistic map (lo) and its attractor, as a function of the bifurcation parameter *μ*, for different values of $\ell$. (**b**) The same data under rescaling (decreasing the density of points, for clarity sake), together with data from the transcritical bifurcation in normal form (tc) and the saddle-node bifurcation (sn). The collapse of the curves into a single one validates the scaling law, Eq. (4), and its universal character. The scaling function is in agreement with *G*(−|*y*|). Note that the initial condition *x*_0_ is taken uniformly randomly between 0.25 and 0.75, which is inside the range necessary for all the iterations to be above the fixed point. This range is, below the bifurcation point, 0 < *x*_0_ < 1 (lo), 0 < *x*_0_ < 1 + *μ* (tc), and, above, 1 − *μ*^−1^ < *x*_0_ < *μ*^−1^ (lo), *μ* < *x*_0_ < 1 (tc), $\sqrt{\mu } < {x}_{0} < 1-\sqrt{\mu }$ (sn).

The scaling law (4) means that any attractor of a one-dimensional map is approached in the same universal way, as long as a Taylor expansion as the one in Eq. (2) holds, in particular if *f* ″(*q*) ≠ 0. In this sense one may talk about a “universality class”, as displayed in Fig. 1. The idea is that for each value of the number of iterations $\ell$ one has to pick a value of *M* (which depends on the parameters of *f*(*x*)) for which $z=\ell (M-1)$ (the rescaled difference concerning the point *M* = 1) remains constant. Note that, in order to have a finite *z*, as $\ell$ is large, *M* = *f* ′(*q*) will be close to 1, implying that the system will be close to its bifurcation point, corresponding to *M* = 1 (where the attractive fixed point will lose its stability). Therefore, in the scaling law, *C* can be replaced by its value at the bifurcation point ${C}_{\ast }$, so, we write $C={C}_{\ast }$ in Eq. (4).

In principle, the value of the initial condition *x*_0_ is not of fundamental importance. The same results can be obtained, for example, by taking *x*_1_ = *f*(*x*_0_) as the initial condition and then replacing $\ell$ by $\ell -1$ because, for very large $\ell$, $\ell \simeq \ell -1$. Therefore, as $\ell$ grows, memory of the initial condition is erased, as $\ell$ can be made as large as desired. However, *x*_0_ has to fulfill *x*_0_ < *q* if ${C}_{\ast } > 0$ and *x*_0_ > *q* if ${C}_{\ast } < 0$, in the same way that all the iterations *x*_*n*_ must also satisfy these inequalities (i.e., all the iterations have to be on the same “side” of the point *q*, see the caption of Fig. 1 for the concrete conditions). The scaling law implies that plotting $[q-{f}^{\ell }({x}_{0})]{C}_{\ast }\ell$ as a function of $\ell (M-1)$ must yield a data collapse of the curves corresponding to different values of $\ell$ onto the scaling function *G*.

For example, for the logistic (lo) map^20^, *f*(*x*) = *f*_*lo*_(*x*) = *μx*(1 − *x*), a transcritical bifurcation takes place at *μ* = 1 and the attractor is at *q* = 0 for *μ* ≤ 1 and at *q* = 1 − 1/*μ* for *μ* ≥ 1, which leads to $${M}_{lo}={f}_{lo}^{^{\prime} }(q)=\mu $$ for *μ* ≤ 1 and *M*_*lo*_ = 2 − *μ* for *μ* ≥ 1, and also to $${C}_{lo\ast }=-\,1$$. Therefore, $$z=\ell (M-1)=-\,\ell |\mu -1|$$ and $${f}_{lo}^{\ell }({x}_{0})-q\simeq {\ell }^{-1}$$$$G(-\ell |\mu -1|)$$ for *x*_0_ > *q*. Thus, in order to verify the collapse of the curves onto the function *G*, the quantity $$[\,{f}_{lo}^{\ell }({x}_{0})-q]\ell $$ must be displayed as a function of −$$\ell |\mu -1|$$; if the resulting plot does not change with the value of $$\ell $$ the scaling law can be considered to hold. Alternatively, the two regimes $$\mu \,\gtrless \,1$$, can be observed by writing $$[\,{f}_{lo}^{\ell }({x}_{0})-q]\ell $$ as a function of $$y=\ell (\mu -1)$$. In the latter case the scaling function turns out to be *G*(−|*y*|). Figure 1(b) shows precisely this; the nearly perfect data collapse for large $$\ell $$ is the indication of the fulfillment of the finite-time scaling law. For comparison, Fig. 1(a) shows the same data with no rescaling (i.e., just the distance to the attractor as a function of the bifurcation parameter *μ*). In the case of the normal form of the transcritical (tc) bifurcation (in the discrete case), *f*_*tc*_(*x*) = (1 + *μ*)*x* − *x*^2^, the bifurcation takes place at *μ* = 0 (with *q* = 0 for *μ* ≤ 0 and *q* = *μ* for *μ* ≥ 0). This leads to exactly the same behavior for $$z=-\,\ell |\mu |$$ (or for $$y=\ell \mu $$ in order to separate the two regimes, as shown overimposed in Fig. 1(b), again with very good agreement).

For the saddle-node (sn) bifurcation (also called fold or tangent bifurcation^31^), in its normal form (discrete system), *f*_*sn*_(*x*) = *μ* + *x* − *x*^2^, the attractor is at $q=\sqrt{\mu }$ (only for *μ* > 0), so the bifurcation is at *μ* = 0, which leads to ${M}_{sn}=1-2\sqrt{\mu }$ and ${C}_{sn\ast }=-\,1$. The scaling law can be written as

5 $${f}_{sn}^{\ell }({x}_{0})-\sqrt{\mu }\simeq \frac{1}{\ell }G(\,-\,2\ell \sqrt{\mu }).$$

To see the data collapse onto the function *G* one must represent $[{f}_{sn}^{\ell }({x}_{0})-\sqrt{\mu }]\ell$ as a function of $z=-\,2\ell \sqrt{\mu }$ (or as a function of *y* = −*z* for clarity sake, as shown also in Fig. 1(b)). In order to create a horizontal axis that is linear in *μ*, we first define $z=-\,\sqrt{u}$, in which case ${f}_{sn}^{\ell }({x}_{0})-\sqrt{\mu }\simeq F(4{\ell }^{2}\mu )/\ell$, with a transformed scaling function $F(u)=G(\,-\,\sqrt{u})=\sqrt{u}/({e}^{\sqrt{u}}-1),$ and then use $u=-\,{z}^{2}=4{\ell }^{2}\mu$ for the horizontal axis of the rescaled plot.

Although the key idea of the finite-time scaling law, Eq. (4), is to compare the solution of the system at “corresponding” values of $$\ell $$ and *μ* (such that *z* is constant, in a sort of law of corresponding states^21^), the law can also be used at fixed *μ*. At the bifurcation point (*μ* = *μ*_*c*_, so *z* = 0), we find that the distance to the attractor decays hyperbolically, i.e., $$|\,{f}^{\ell }({x}_{0})-q|=|{C}_{\ast }\ell {|}^{-1}$$, as it is well known, see for instance ref.^19^. Out of the bifurcation point, for non-vanishing *μ* − *μ*_*c*_ we have *z* → −∞ (as $$\ell \to \infty $$) and then *G*(*z*) → *e*^−*z*^, which leads to $${f}^{\ell }({x}_{0})-q\simeq {\ell }^{-1}$$$${e}^{-z}\simeq {e}^{-\ell /\tau }$$, where, from the expression for *z*, we find that the characteristic time *τ* diverges as *τ* = 1/|*μ* − *μ*_*c*_| for the transcritical bifurcation (both in normal form and in the logistic form) and as $$\tau =1/(2\sqrt{\mu -{\mu }_{c}})$$ for the saddle-node bifurcation (with *μ*_*c*_ = 0 in the normal form)^12^. These laws, mentioned in the introduction, have been reported in the literature as scaling laws^20^, but in order to avoid confusion we propose calling them power-law divergence laws, and keep the term scaling law for behaviors such as those in Eqs (1), (4) and (5). Note that this sort of law arises because *G*(*z*) is asymptotically exponential for *z* → −∞; in contrast, the equivalent of *G*(*z*) in the equation of state of a magnetic system in the thermodynamic limit is a power law, which leads to the Curie-Weiss law^32^.

## Scaling Law for the Distance to the Fixed Point at Bifurcation in the Transcritical Bifurcation

In some cases, the distance between ${f}^{\ell }({x}_{0})$ and some constant value of reference will be of more interest than the distance to the attractive fixed point *q*, as the value of *q* may change with the bifurcation parameter. For the transcritical bifurcation we have two fixed points, *q*_0_ and *q*_1_, and they collide and interchange their character (attractive to repulsive, and vice versa) at the bifurcation point. It will be assumed that *q*_0_ is constant independent of the bifurcation parameter (naturally, *q*_1_ will not be constant), and that “below” the bifurcation point *q*_0_ is attractive and *q*_1_ is repulsive, and vice versa “above” the bifurcation. We will be interested in the distance between *q*_0_ and ${f}^{\ell }({x}_{0})$, i.e., ${q}_{0}-{f}^{\ell }({x}_{0})$, which, below the bifurcation point corresponds to the quantity calculated previously in Eq. (4), but not above. The reason is that, there, *q* was an attractor, but now *q*_0_ can be attractive or repulsive. Note that, without loss of generality, we can refer ${q}_{0}-{f}^{\ell }({x}_{0})$ as the distance of ${f}^{\ell }({x}_{0})$ to the “origin”.

Following ref.^29^, we seek a relationship between both fixed points when the system is close to the bifurcation point. As, in that case, ${q}_{1}\simeq {q}_{0}$, we can expand *f*(*q*_1_) around *q*_0_ to obtain

$$f({q}_{1})={q}_{1}={q}_{0}+{M}_{0}({q}_{1}-{q}_{0})+{C}_{0}{({q}_{1}-{q}_{0})}^{2}+{\mathscr{O}}{({q}_{1}-{q}_{0})}^{3},$$

which leads directly to

6 $${M}_{0}-1={C}_{0}({q}_{0}-{q}_{1}),$$

to the lowest order in (*q*_1_ − *q*_0_). Naturally, *M*_0_ = *f* ′(*q*_0_) and *C*_0_ = *f* ″(*q*_0_)/2. We also seek a relationship between *M*_1_ = *f* ′(*q*_1_) and *M*_0_. Expanding *f* ′(*q*_1_) around *q*_0_, $f^{\prime} ({q}_{1})={M}_{1}={M}_{0}+2{C}_{0}({q}_{1}-{q}_{0})+{\mathscr{O}}{({q}_{1}-{q}_{0})}^{2},$ which, using Eq. (6), leads to

7 $${M}_{0}-1=1-{M}_{1},$$

to the first order in (*q*_1_ − *q*_0_).

We now write ${q}_{0}-{f}^{\ell }({x}_{0})={q}_{0}-{q}_{1}+{q}_{1}-{f}^{\ell }({x}_{0})$. For *q*_0_ − *q*_1_ we will apply Eq. (6), and for ${q}_{1}-{f}^{\ell }({x}_{0})$ we can apply Eq. (4), as *q*_1_ is of attractive nature “above” the bifurcation point; then

$${q}_{0}-{f}^{\ell }({x}_{0})\simeq \frac{{M}_{0}-1}{{C}_{0}}+\frac{1}{{C}_{1}\ell }G(\ell ({M}_{1}-1)),$$

(with *C*_1_ = *f* ″(*q*_1_)/2), and defining $y=\ell ({M}_{0}-1)$ we obtain (with the form of the scaling function, Eq. (3)),

$${q}_{0}-{f}^{\ell }({x}_{0})\simeq \frac{y}{{C}_{0}\ell }+\frac{1}{{C}_{1}\ell }(\frac{z{e}^{z}}{{e}^{z}-1}).$$

Using Eq. (7) it can be shown that

8 $$z=\ell ({M}_{1}-1)=-\,\ell ({M}_{0}-1)=-\,y,$$

(so, the *y* introduced here is the same *y* introduced in the previous section), and therefore,

$${q}_{0}-{f}^{\ell }({x}_{0})\simeq \frac{1}{{C}_{\ast }\ell }(y+\frac{-y{e}^{-y}}{{e}^{-y}-1})=\frac{1}{{C}_{\ast }\ell }\frac{y{e}^{y}}{{e}^{y}-1},$$

where we have also used that ${C}_{1}={C}_{0}={C}_{\ast }$, to the lowest order, with ${C}_{\ast }$ being the value at the bifurcation point. Therefore, we obtain the same scaling law as in the previous section:

9 $${q}_{0}-{f}^{\ell }({x}_{0})\simeq \frac{1}{{C}_{\ast }\ell }G(y),$$

with the same scaling function *G*(*y*) as in Eq. (3), although the rescaled variable *y* is different here (*y* ≠ *z*, in general). This is possible thanks to the property *y* + *G*(−*y*) = *G*(*y*) that the scaling function satisfies. Note that the scaling law (1) has the same form as the finite-time scaling (9) with *y* given by Eq. (8), and therefore we can identify *β* = *ν* = 1. Note also that we can identify *M*_0_ = *f* ′(*q*_0_) with a bifurcation parameter, as it is *M*_0_ < 1 “below” the bifurcation point (*M*_0_ = 1) and *M*_0_ > 1 “above”. In fact, *M*_0_ can be considered as a natural bifurcation parameter, as the scaling law (4) expressed in terms of *M*_0_ becomes universal. *M* defined in the previous section cannot be a bifurcation parameter as it is never above one because it is defined with respect to the attractive fixed point.

**Figure 2:**
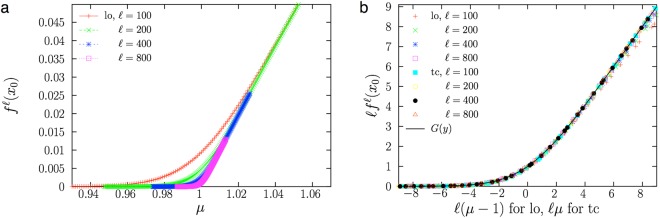
(**a**) $\ell$–th iteration of the logistic map as a function of the bifurcation parameter *μ*, for different values of $\ell$. Same initial conditions as in previous figure. (**b**) Same data under rescaling (decreasing density of points), plus analogous data coming from the transcritical bifurcation in normal form. The data collapse shows the validity of the scaling law, Eq. (9), with scaling function *G*(*y*) from Eq. (3).

For the transcritical bifurcation of the logistic map we identify *q*_0_ = 0 and *M*_0_ = *μ*, so $y=\ell (\mu -1)$. For the normal form of the transcritical bifurcation, *q*_0_ = 0 but *M*_0_ = *μ* + 1, so $y=\ell \mu$. Consequently, Fig. 2(a) shows ${f}^{\ell }({x}_{0})$ (the distance to *q*_0_ = 0) as a function of *μ*, for the logistic map and different $\ell$, whereas Fig. 2(b) shows the same results under the corresponding rescaling, together with analogous results for the normal form of the transcritical bifurcation. The data collapse supports the validity of the scaling law (9) with scaling function given by Eq. (3).

## Scaling Law for the Iterated Value *x*_*n*_ in the Saddle-Node Bifurcation

**Figure 3:**
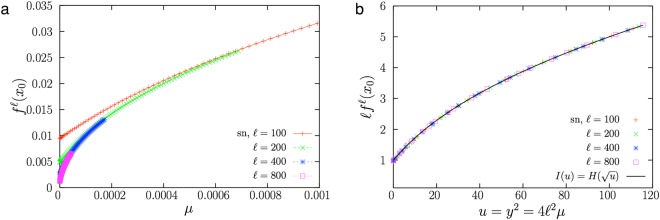
(**a**) Same as Fig. 2(a) but for the saddle-node bifurcation in normal form. (**b**) Rescaling of the same data (with decreased density of points). The data collapse supports the scaling law and the scaling function *I*(*u*) given by Eq. (10).

In the case of a saddle-node bifurcation, the $\ell$–th iterate can be isolated from Eq. (5) to obtain

$${f}^{\ell }({x}_{0})\simeq \frac{1}{\ell }[\frac{2\ell \sqrt{\mu }}{2}+G(-2\ell \sqrt{\mu })]=\frac{1}{\ell }H(y)$$

with $y=-\,z=2\ell \sqrt{\mu }$ and *H*(*y*) = *y*(*e*^*y*^ + 1)(*e*^*y*^ − 1)^−1^/2. Therefore, the representation of $\ell {f}^{\ell }({x}_{0})$ as a function of $2\ell \sqrt{\mu }$ unveils the shape of the scaling function *H*. In terms of $u={y}^{2}=4{\ell }^{2}\mu$,

10 $${f}^{\ell }({x}_{0})\simeq \frac{1}{\ell }I(u),\,{\rm{with}}\,I(u)=H(\sqrt{u})=\frac{\sqrt{u}}{2}\frac{({e}^{\sqrt{u}}+1)}{({e}^{\sqrt{u}}-1)},$$

and, therefore, plotting $\ell {f}^{\ell }({x}_{0})$ as a function of $4{\ell }^{2}\mu$ must lead to the collapse of the data onto the scaling function *I*(*u*), as shown in Fig. 3. Comparison with the finite-size scaling law (1) allows one to establish *β* = *ν* = 1/2 for this bifurcation (and bifurcation parameter *μ*, not $\sqrt{\mu }$).

## Conclusions

By means of scaling laws, we have made a clear analogy between bifurcations and phase transitions^23^, with a direct correspondence between, on the one hand, the bifurcation parameter, the bifurcation point, and the finite-time solution ${f}^{\ell }({x}_{0})$, and, on the other hand, the control parameter, the critical point, and the finite-size order parameter. However, in phase transitions, the sharp change of the order parameter at the critical point arises in the limit of infinite system size; in contrast, in bifurcations, the sharpness at the bifurcation point shows up in the infinite-time limit, $\ell \to \infty$. So, finite-size scaling in one case corresponds to finite-time scaling in the other. Specifically, we conclude that the finite-size scaling behavior derived in ref.^29^ can be directly understood from the transcritical bifurcation underlying the Galton-Watson branching process. It is remarkable that the critical behavior of such a stochastic process is governed by a bifurcation of a deterministic dynamical system.

Moreover, by using numerical simulations we have tested that the finite-time scaling laws also hold for dynamical systems continuous in time, as well as for the pitchfork bifurcation in discrete time, although with different exponents and scaling function in this case (this is due to the fact that the condition *f* ″(*q*) ≠ 0 does not hold). The use of the finite-time scaling concept by other authors does not correspond with ours. For instance, although ref.^33^ presents a scaling law for finite times, the corresponding exponent *ν* there turns out to be negative, which is not in agreement with the genuine finite-size scaling around a critical point. In addition, we have also been able to derive the power-law divergence of the transient time to reach the attractor out of criticality^12,19,20^.

Our results could be useful for interpreting different types of fixed points found in renormalization group theory^23^. Also, they might allow to idenfity the type of bifurcations in systems for which information is limited to short transients, such as in ecological systems. In this way, the scaling relations established in this article could be used as warning signals^34^ to anticipate the nature of collapses or changes in ecosystems^5,6,34–36^ (due to, e.g., transcritical or saddle-node bifurcations) and in other dynamical systems suffering shifts.
